# Phylogenomic Perspective on a Unique *Mycobacterium bovis* Clade Dominating Bovine Tuberculosis Infections among Cattle and Buffalos in Northern Brazil

**DOI:** 10.1038/s41598-020-58398-5

**Published:** 2020-02-04

**Authors:** Marília Lima da Conceição, Emilyn Costa Conceição, Ismari Perini Furlaneto, Sandro Patroca da Silva, Arthur Emil dos Santos Guimarães, Pedro Gomes, María Laura Boschiroli, Lorraine Michelet, Thomas Andreas Kohl, Katharina Kranzer, Loreno da Costa Francez, Luana Nepomuceno Gondim Costa Lima, Isabel Portugal, João Perdigão, Karla Valéria Batista Lima

**Affiliations:** 1 State University of Pará, Belém, Brazil; 2 Evandro Chagas Institute, Anandideua, Brazil; 3 Research Institute for Medicines (iMed.ULisboa), Faculty of Pharmacy, Universidade de Lisboa, Lisbon, Portugal; 4 Federal University of Rio de Janeiro, Rio de Janeiro, Brazil; 5 Oswald Cruz Foundation, Rio de Janeiro, Brazil; 6 French Agency for Food, Environmental and Occupational Health and Safety, Maisons-Alfort, France; 7 Leibniz Research Center Borstel, Borstel, Germany; 8 German Center for Infection Research (DZIF), Heidelberg, Germany; 9 Research Center Borstel Leibniz Lung Center, National Reference Center for Mycobacteria, Borstel, Germany; 10 London School of Hygiene and Tropical Medicine, London, UK London School of Hygiene and Tropical Medicine, Clinical Research Department, London, UK; 11 Federal Rural University of Amazon, Belém, Brazil

**Keywords:** Applied microbiology, Microbial genetics

## Abstract

Lack of routine surveillance in countries endemic for bovine tuberculosis (TB) and limited laboratory support contributes to the inability to differentiate the *Mycobacterium tuberculosis* Complex species, leading to an underestimated burden of the disease. Here, Whole-Genome Sequencing of *Mycobacterium bovis* isolated from tissues with TB-like lesions obtained from cattle and buffalos at Marajó Island, Brazil, demonstrates that recent transmission of *M. bovis* is ongoing at distinct sites. Moreover, the *M. bovis* epidemiology in this setting is herein found to be dominated by an endemic and unique clade composed of strains evolved from a common ancestor that are now genetically differentiated from other *M. bovis* clades. Additionally, envisioning a rapid strain differentiation and tracing across multiple settings, 28 globally validated strain-specific SNPs were identified, three of which considered as robust markers for the *M. bovis* Marajó strain. In conclusion, this study contributes with data regarding the identification of a novel *M. bovis* phylogenetic clade responsible for ongoing transmission events in both cattle and buffalo species in Brazil, provides a framework to investigate the dissemination of this highly prevalent strain and, holds the potential to inform TB control strategies that may help to prevent the spread of bovine and zoonotic TB.

## Introduction

Tuberculosis (TB) is a worldwide important infectious disease in humans and other animals resulting in substantial morbidity and mortality caused by the *Mycobacterium tuberculosis* Complex (MTBC) pathogens^1,2^. Among these, *Mycobacterium bovis* is the main etiological agent of bovine TB (bTB) in herds and is associated with a decreased livestock productivity due to early disposal of animals of high zootechnical value with subsequent economic impact^3^. The impact of bTB and *M. bovis* is not only restricted to economic aspects of livestock production. Although the economic losses can ascend to, e.g., US$18 200 in a single region as reported for Makurdi in Nigeria due to a prevalence 1.9% among 61 654 slaughtered cattle^4^, zoonotic TB is still a major public health problem as it is estimated to cause 140 000 new cases and more than 12 000 deaths in humans worldwide^5^.

An important factor for the control of zoonotic TB is that human TB caused by *M. bovis* is possibly underestimated^6^. In fact, combating zoonotic TB is a goal of WHO’s End TB Strategy since the lack of scientific attention to this problem warrants further studies, especially in areas where bTB remains endemic. Besides lack of routine surveillance data to differentiate *M. bovis* from *M. tuberculosis sensu stricto* in humans, the burden of the disease in humans is unknown, often showing uncommon clinical presentations, and a possible cause of treatment failure due to *M. bovis* intrinsic resistance to pyrazinamide (PZA), an important first-line drug^5^. The latter is of special concern as it may pose a stepping-stone to the already reported human *M. bovis* infections by multidrug-resistant strains^7^.

The mere presence of *M. bovis* among cattle warns of the zoonotic risk to humans, especially those living at the human-animal interface. In the northern region of Brazil, the Marajó Island in the State of Pará, an unusual insular system, harbours the largest buffalo herd at a nationwide scale (over 520,000 head)^8^. No data presently exists regarding notification rates of bTB in this island, but previous evidence supports the presence of *M. bovis* in cattle and buffalo with biopsy specimens suggestive of TB^9^.

While the body of knowledge concerning the distribution of *M. tuberculosis sensu stricto* strains is already well understood in most settings, molecular data and phylogeny of the *M. bovis* ecotype are scarce^10^. Envisioning strain differentiation, genotyping schemes and molecular methods were developed to discriminate MTBC clinical samples, such as spoligotyping and MIRU-VNTR (Mycobacterial Interspersed Repetitive Units - Variable Number of Tandem Repeats). However, these methods have limitations for phylogenetic studies due to a low discriminatory power or, *e.g*., an existing propensity of molecular markers for convergent evolution, leading to identical or similar patterns in strains that are phylogenetically and epidemiologically unrelated^11,12^. In this aspect, the advances made over the last decade on high-throughput sequencing technology now enable a comprehensive access to the entire genomic information of any given strain^13^.

Herein, to elucidate the genetic clonality and transmission dynamics of *M. bovis*, we have employed classical genotyping methods, along with state-of-the-art genome-wide phylogenetic reconstruction to evaluate the genomic clustering among cattle and buffalo from an abattoir in the Marajó Island while simultaneously providing a global phylogenetic context for these strains.

## Results

### bTB in the Marajo Island, Brazil, and *M. bovis* genotypic diversity

The initial approach to investigate the clonality and population structure of *M. bovis* in this setting relied on the use of classical typing methods (spoligotyping and MIRU-VNTR) in the characterization of 22 *M. bovis* isolates from nine cattle and nine buffalos from October 2014 to December 2015. The genotypic characterization by spoligotyping yielded two distinct profiles (Table 1): the predominant spoligotype was SB0822/SIT997, detected in 21 isolates, which is characterized by the absence of spacers 3, 4, 9, 16, and 39 to 43; the remaining isolate corresponded to the spoligotype SB0885/SIT986, which lacks spacers 3, 4, 5, 6, 9, 16 and 39 to 43.

**Table 1:** *Mycobacterium bovis* isolates from cattle and buffalos in the Marajó Island (Pará, Brazil). For each isolate, the year of isolation, city of origin along with classical genotyping data and mapping statistics are shown.

Isolate	Year	Infection site	Animal	City	Genotype		Mapping Statistics	
					SIT/SB^b^	MIRU-VNTR cluster^c^	No. of Mapped Reads(%)	Mean Coverage Depth
G00001	2014	Lymph nodes	Buffalo 1	Soure	997/0822	1	2915368 (59.0)	85.3582
G00002^a^	2014	Lymph nodes	Buffalo 2	Soure	997/0822	4	1217486 (39.6)	34.8304
G00003^a^	2015	Lymph nodes	Cattle 1	Chaves	997/0822	—	443667 (10.7)	7.7398
G00004	2015	Lymph nodes	Cattle 2	Chaves	997/0822	1	3889617 (98.8)	114.7544
G00005	2015	Esophagus	Cattle 3	Chaves	997/0822	1	4452372 (99.7)	131.8627
G00006	2015	Lymph nodes	Cattle 4	Chaves	997/0822	1	3166441 (99.7)	93.1683
G00007	2015	Lymph nodes	Cattle 5	Chaves	997/0822	1	3392896 (99.6)	100.1515
G00008^a^	2015	Abomasum	Cattle 6	Chaves	997/0822	1	796095 (18.7)	17.0591
G00009	2015	Esophagus	Buffalo 3	Santa Cruz do Arari	997/0822	1	3403743 (98.2)	100.0624
G00010	2015	Liver	Buffalo 4	Soure	997/0822	2	3847522 (99.7)	115.2658
G00011	2015	Lymph nodes	Buffalo 5	Soure	997/0822	1	3117214 (98.8)	91.9547
G00012	2015	Rumen	Cattle 7	Soure	997/0822	1	2873682 (75.6)	84.5041
G00013	2015	Lymph nodes	Buffalo 6	Soure	997/0822	3	4823761 (99.2)	143.5417
G00014	2015	Lymph nodes	Buffalo 7	Soure	997/0822	1	4544806 (98.8)	135.971
G00015	2015	Lymph nodes	Cattle 8	Soure	986/0885	1	5509443 (98.5)	166.1321
G00016^a^	2015	Lymph nodes	Cattle 9	Soure	997/0822	—	779376 (14.3)	13.4106
G00017	2015	Lymph nodes	Buffalo 8	Soure	997/0822	3	5174290 (99.9)	155.8405
G00018	2015	Lymph nodes	Buffalo 9	Cachoeira do Arari	997/0822	5	4438288 (99.8)	131.5516
G00019	2015	Liver	Cattle 3	Chaves	997/0822	1	4254501 (99.9)	124.9458
G00020^a^	2015	Lymph nodes	Cattle 7	Soure	997/0822	2	729323 (13.7)	15.6735
G00021	2015	Abomasum	Cattle 6	Chaves	997/0822	1	16287213 (98.6)	476.0113
G00022	2015	Lymph nodes	Cattle 7	Soure	997/0822	1	15229721 (96.0)	440.3858

^a^Genomes excluded due to low coverage depth and/or low percentage of mapping reads;

^b^Shared International Type and SB-number according to SITVIT2 and *Mycobacterium bovis* Spoligotyping Database (MBovis.org);

^c^24-loci MIRU-VNTR profiles: 1 (225322341363454251322312); 2 (225322341363454251322312); 3 (225322341362454251322312); 4 (225322141363474251322312); and 5 (225322341363474251322312).

Comparing both profiles, one can speculate that the SB0822/SIT997 profile is the parental strain with the isolate belonging to SB0855/SIT986 type being a derived strain that underwent diversification at the DR locus by losing the 5^th^ and 6^th^ spacer regions as this is the only difference between these isolates and is coherent with the unidirectional evolution observed at the DR locus^12^.

To further support this notion, and contrary to spoligotype diversity, 24-loci MIRU-VNTR of 20 *M. bovis* isolates recovered from 17 animals (two isolates/animals were excluded as these failed to amplify more than 10 MIRU-VNTR loci after multiple attempts) revealed a more diverse scenario composed of five profiles. Only VNTR loci 2165 (ETRA), 2461 (ETRB), 1644 (MIRU 16), and 2347 (Mtub29) presented allelic diversity amongst the isolates with a total of three clusters herein detected encompassing a total of 18 isolates.

**Figure 1:**
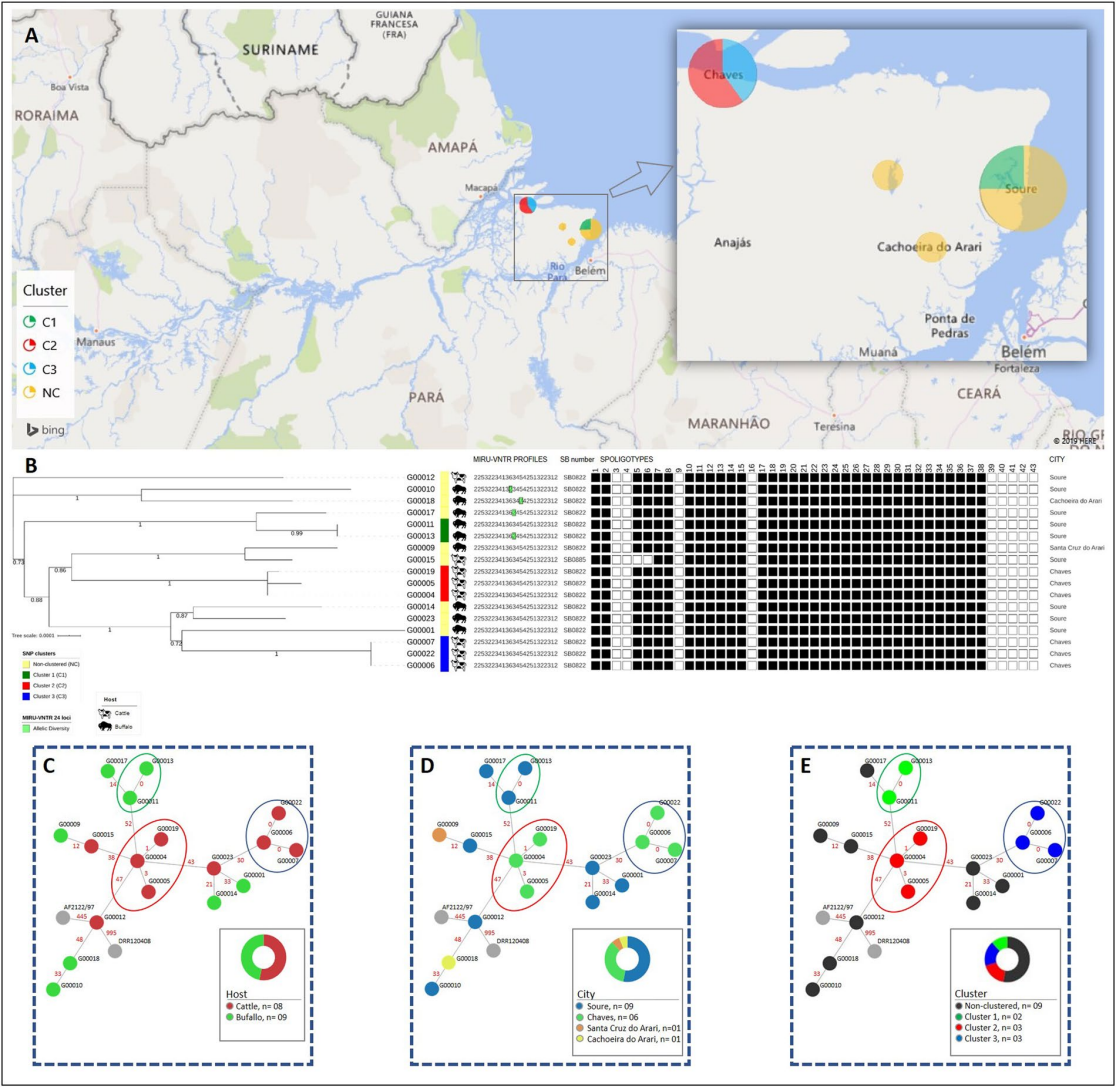
Geographical distribution of genomic clusters, maximum-likelihood phylogenetic and minimum spanning trees (MSTs) of the *M. bovis* isolates from the Marajó Island, Brazil. The geographic distribution of the three genomic clusters found in the Marajó Island shows that *M. bovis* transmission is ongoing at multiple cities in the island (**A**) as a result of clonal expansion of a unique *M. bovis* clade that has disseminated across both cattle and buffalo species (**B**). MSTs with node coloring according to host species (**C**), city of origin (**D**) and genomic cluster (**E**) also support the dissemination of this strain across multiple cities and host species with moderate diversification observed mostly between genomic clusters. The maximum-likelihood phylogenetic tree based on 1773 high-quality genome-wide SNPs (**B**) is shown annotated with the genomic clusters (see legend), host species, MIRU-VNTR and spoligotyping profiles and city of origin of the animal. MIRU-VNTR profiles are represented in a 24-digit numeric string where each-digit represents the number of repeats at a particular locus according to the following order of the loci: 154, 424, 577, 580, 802, 960, 1644, 1955, 2059, 2163b, 2165, 2347, 2401, 2461, 2531, 2687, 2996, 3007, 3171, 3192, 3690, 4052, 4156, 4384 and 4348. Numbers annotated in links between the nodes of the MSTs (**C**–**E**) represent the number of segregating SNP sites between nodes. Figure generated using the Interactive Tree of Life v5 online tool (available at https://itol.embl.de), Microsoft PowerPoint 2016 (Version 1707) and Microsoft Excel 2016 (Version 1707), incl. Microsoft Power Map 3D Data Visualization Tool (https://products.office.com/en-us/business/office) and Phyloviz v2.0 (available at https://online2.phyloviz.net).

The most common MIRU-VNTR profile was shared by 14 isolates, the second and third most common MIRU-VNTR profiles were shared by two isolates and, two isolates exhibited unique profiles and were therefore classified as non-clustered strains (Table 1). Noteworthy, under the 24-loci MIRU-VNTR typing method, the single SB0855/SIT986 clinical isolate was clustered in the largest MIRU-VNTR cluster, mostly comprised by SB0822/SIT997 isolates (Table 1 and Fig. 1).

**Figure 2:**
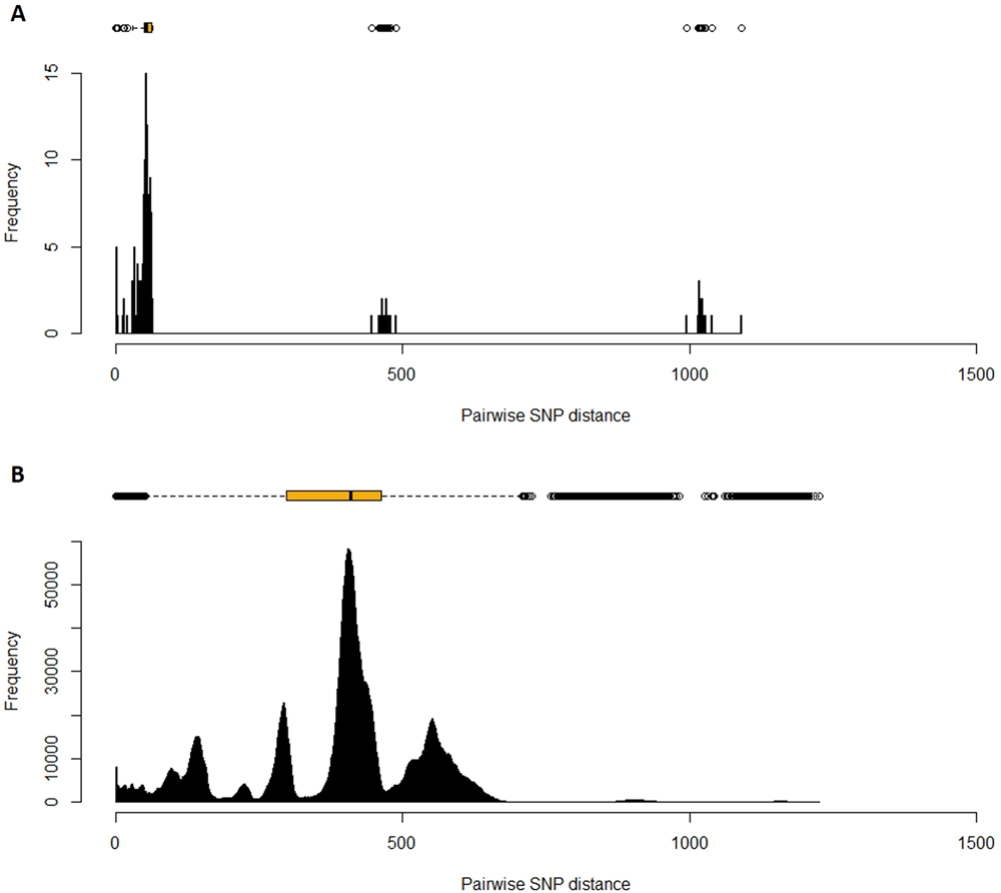
Frequency histogram of SNP pairwise distances across the Marajó Island *M. bovis* isolates (**B**) and across the global *M. bovis* dataset (**B**). Pairwise distance distribution across the Marajó *M. bovis* isolates (**A**) show a restricted distribution when compared with the global dataset (**B**) with the strains isolated from the Marajó Island showing a SNP pairwise distance of up to 64 SNPs. In panel A, two additional distance peaks around 450 and 1050 bp represent the distance of the Marajó strains towards *M. bovis* AF2122/97 and *M. caprae* EPDC01, which were used as reference for mapping and to root the phylogenetic trees, respectively.

The latter lends support to the previous notion that SB0855/SIT986 is a likely divergent strain from the predominant SB0822/SIT997 strains detected in the Marajo Island (Fig. 2). Overall, both MIRU-VNTR and spoligotyping suggest that a highly clonal *M. bovis* population structure exists in the Marajó Island albeit slightly more diverse under a MIRU-VNTR perspective as would already be expected given its superior discriminatory power^14,15^.

### Genomic diversity of *M. bovis* in the Marajó Island reveals the Marajó *M. bovis* strain as a unique monophyletic branch within *M. bovis*

Out of the 22 *M. bovis* isolates sequenced, 17 genomes were retained for downstream genome-wide phylogenetic analysis after genome quality control. These isolates originated from the four municipalities: Soure (n = 9), Chaves (n = 6), Cachoeira do Arari (n = 1) and Santa Cruz do Arari (n = 1) (Table 1).

Search for drug resistance-conferring mutations using TB-Profiler^16^ showed that all isolates were genotypically susceptible to all anti-TB drugs except PZA due to the *M. bovis* specific H57D missense mutation in the *pncA* gene of *M. bovis*^17^.

Upon phylogenetic analysis, the Marajó Island *M. bovis* isolates were found to comprise a monophyletic clade composed of isolates within a maximum pairwise distance of 64 SNPs and is herein referred as the “Marajó-strain” (Fig. 1). These findings are also corroborated by the analysis of the frequency distribution of pairwise SNP distances between isolates from the Marajó Island (0–64 SNPs) which, depending on the isolate are 445–488 and 995–1039 SNPs apart from *M. bovis* AF2122/97 and *M. caprae* EPDC01, respectively (Fig. 2). Also, three genomic clusters (C1–3) harboring isolates within a maximum pairwise distance of 5 SNPs were detected with each cluster being composed of isolates originating from the same city within the Marajó Island and isolated from the same animal species. Moreover, the topology of the reconstructed phylogenetic tree is suggestive of historical dissemination by this strain across both cattle and buffalo species and, at multiple geographical locations in the Marajó Island (Fig. 1).

**Figure 3:**
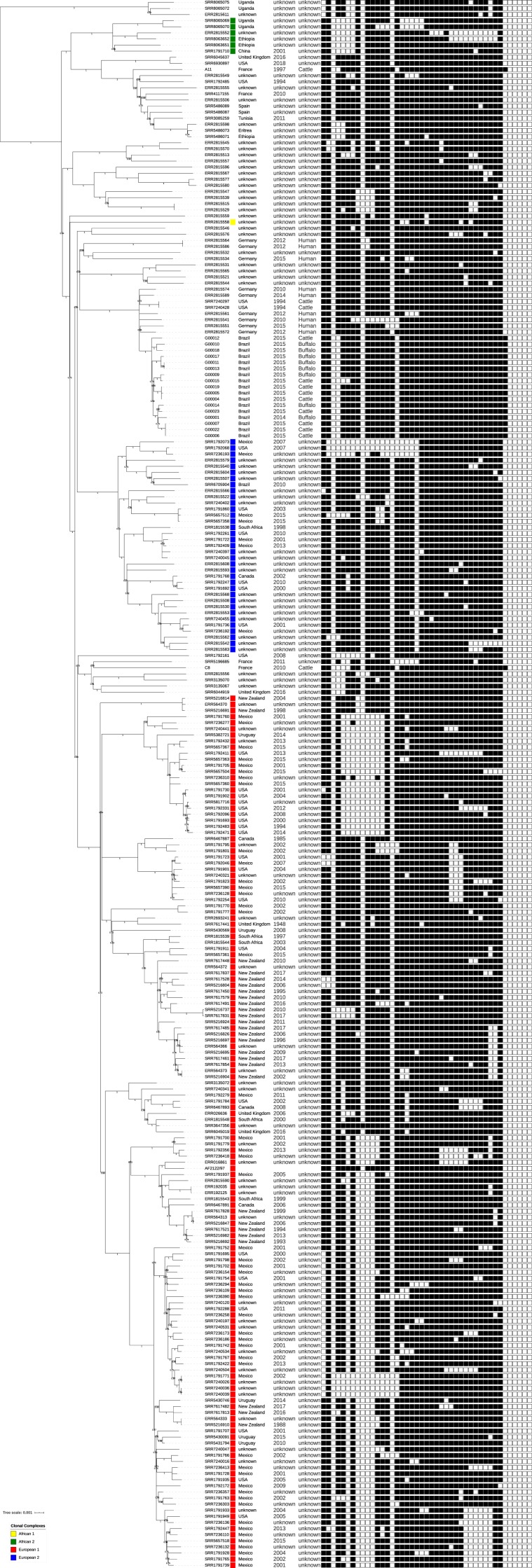
Global phylogenetic tree of 257 *M. bovis* isolates (including the 17 *Marajó M. bovis* isolates) highlighting the monophyletic nature of the *M. bovis* Marajó clade. The tree is shown annotated with the isolate ID or ENA run accession, clonal complex colored according to the legend in the bottom-left corner, country of isolation, year of isolation, host species, and spoligotyping profile (in this order). Figure generated using the Interactive Tree of Life v5 online tool (available at https://itol.embl.de).

Next, in order to provide an adequate evolutionary background for this strain, we compared the SNP pairwise distance between the isolates of our study and a global *M. bovis* dataset comprised of 3 402 *M. bovis* genomes publicly available, along with two genomes obtained from ANSES (Maisons-Alfort, France), isolated in France and belonging to the SB0822 and SB0855 types (Supplementary Table S1; Fig. 2). This global dataset showed an ample and continuous distribution of pairwise SNP distances, congruent with the *M. bovis* global phylogenetic representation of this dataset (Fig. 2). Across these, only eight genomes were found to be within a maximum pairwise distance of 150 SNPs from the Marajó strains. To further investigate the phylogenetic context of the the Marajó strains, a total of 240 *M. bovis isolates*, representative of 150 SB types (along with 18 spoligotyping patterns not found in the *Mycobacterium bovis* Spoligotyping Database) and originating from over 15 countries, were selected for maximum-likelihood phylogenetic analysis (Fig. 3). This sample of 240 *M. bovis* genomes included the SB0822 and SB0885 strains isolated in France with the same spoligotype pattern as the Marajó isolates and the *M. bovis* reference AF2122/97. The phylogenetic tree obtained confirmed the monophyletic nature of the Marajó *M. bovis* isolates in a global context, and, albeit unclassified as per the current rules and genetic markers associated with the different clonal complexes, these strains did form a parallel branch to the European 2 clonal complex^18^ and therefore shared a more recent common ancestor with this specific clade when compared with the other clonal complexes. The French strains, albeit sharing the same spoligotyping profiles, were positioned to distinct *M. bovis* evolutionary branches and were separated from the Marajó strains by 413–492 SNPs. Despite the structural similarity at the DR locus, the phylogenetic positioning and topological structure of the phylogenetic tree denotes genotypic convergence at the DR locus level by distinct and unrelated clades: the Marajó strain in Brazil and the A11 (SB0822) and C8 (SB0885) French isolates. Minimum spanning trees constructed using the goeBURST algorithm also position the Marajó strains as a separate branch close to strains from Germany and among unclassified isolates regarding the *M. bovis* clonal complexes (Supplementary Figures S1 and S2).

### The Marajó *M. bovis* strain comprises a genetically differentiated family

**Figure 4:**
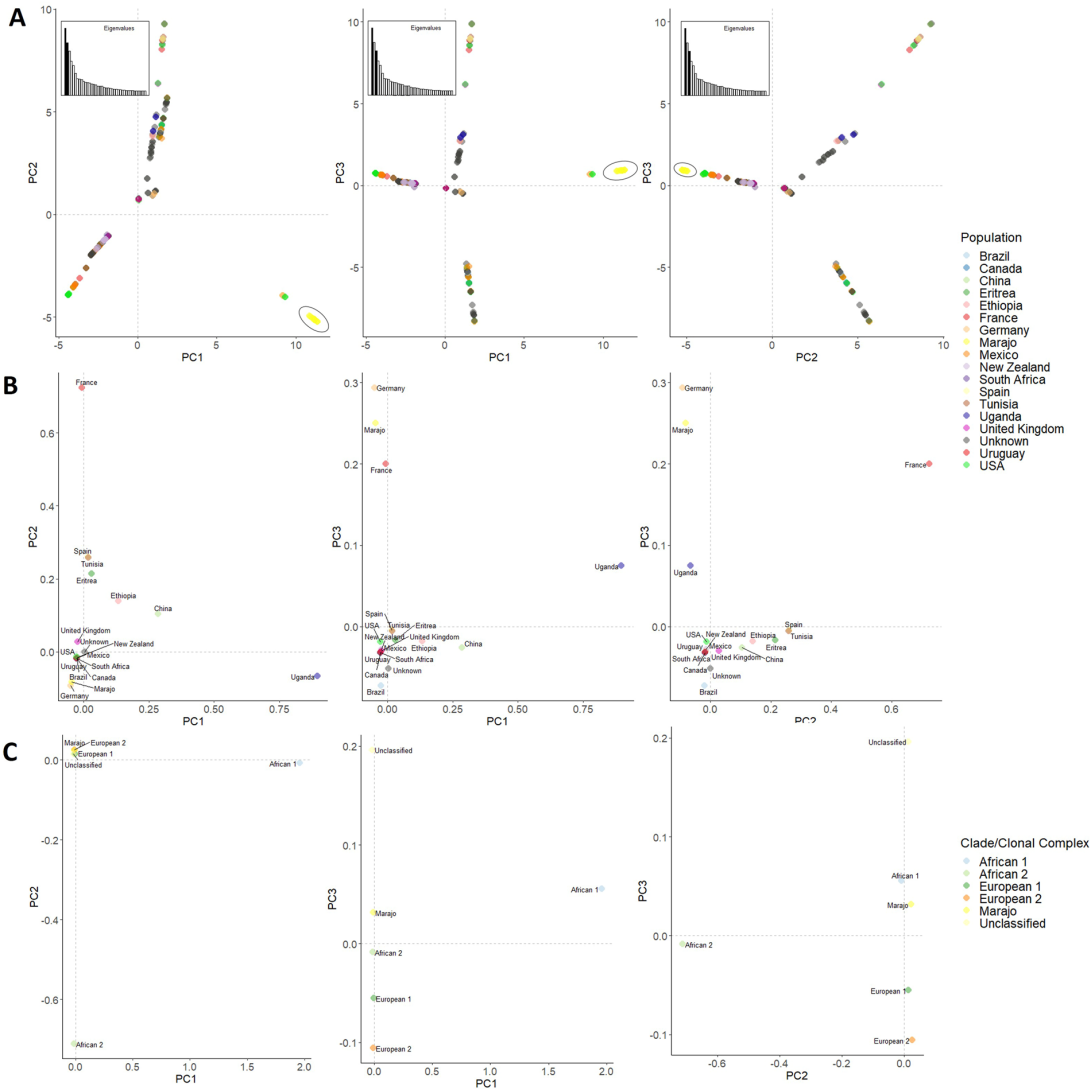
Principal Component Analysis (PCA, panel A) and Principal Coordinate Analysis (PCoA) showing the genetic differentiation of the *M. bovis* Marajó strains upon comparison with additional 240 *M. bovis* isolates (**B** and **C**). PCA demonstrates the clustering of the *M. bovis* Marajó strains across all three principal components while showing the genetic divergence from the remaining isolates (**A**). PCoA corroborates this analysis by demonstrating the unique differentiation of the Marajó *M. bovis* population when compared with other settings (**B**) or with the known and previously described clonal complexes (**C**). Points across the PCA and PCoA plots are coloured according to the study population (country of origin) or clonal complex (see legends).

**Figure 5:**
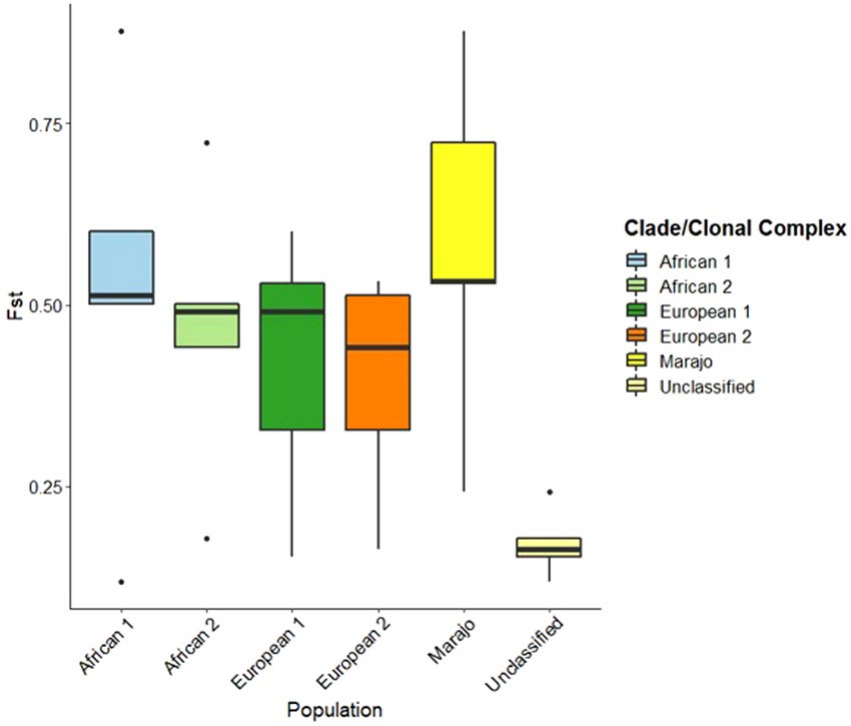
Boxplots of pairwise F_ST_ distances between populations (herein defined as clade or clonal complex), revealing the higher level of genetic differentiation of the Marajó strain when compared with other sub-populations of *M. bovis*.

Given the monophyletic nature of the Marajó strains in a global evolutionary context, we next sough to investigate the genetic differentiation of these strains when compared with the remaining 240 *M. bovis* isolates by using a total of 11 544 core SNPs as variable genetic loci. Principal Component Analysis (PCA) across three main components shows a clear genetic distinctiveness between the Marajó strains and the remaining strains that were herein included for comparative purposes. The PCA also showed that all Marajó strains cluster together across the three main axes and appear to be positioned more closely to isolates from Germany and the United States of America (USA) which, is also compatible with the topology of the phylogenetic tree (Fig. 4). Also, Principal Coordinate Analysis (PCoA) encompassing country of isolation and clonal complex as population descriptives, also do demonstrate that the Marajó strains and the Marajó Island epidemiology is genetically differentiated from other geographical sites and genetic groupings (Fig. 4). This latter notion was also corroborated by pairwise F_ST_ distances calculated between the Marajó strains and the previously described clonal complexes (European 1–2 and African 1–2)^18–21^ which convey a notion of a higher level of genetic differentiation of the Marajó strain when compared with other sub-populations of *M. bovis* (Table 2). In fact, all populations were significantly differentiated although unclassified strains did show a lower level of genetic differentiation, probably owing to its polyphyletic nature (Fig. 5 and Table 2).

**Table 2:** Pairwise F_ST_ distance matrix between different *M. bovis* sub-populations according to Clonal Cluster and comparison with the Marajo clade (unclassified as per the established Clonal Cluster genomic markers). Pairwise F_ST_ values are shown in the matrix upper triangle whereas *p* values are shown in the matrix lower triangle. The pairwise F_ST_ distance values highlight the low genetic differentiation of Unclassified isolates in comparison with other genetic clades, likely owing to its paraphyletic nature, with the Marajo clade showing a high genetic differentiation from the African 1–2 and European 1–2 Clonal Clusters but, lower in comparison with the remaining Unclassified isolates. All comparisons were significant at the 0.05 level of statistical significance.

	African 1	African 2	European 1	European 2	Marajo	Unclassified
African 1		0.501	0.601	0.513	0.877	0.118
African 2	0.000		0.491	0.442	0.723	0.178
European 1	0.000	0.000		0.328	0.529	0.153
European 2	0.000	0.000	0.000		0.533	0.163
Marajo	0.000	0.000	0.000	0.000		0.242
Unclassified	0.001	0.000	0.000	0.000	0.000

### Defining a specific set of SNPs to trace and screen for the Marajó *M. bovis* strain

In order to facilitate rapid strain screening across publicly available genome data and to enable the implementation of laboratory fast tracking of this strain across different settings, we defined a set of specific SNPs that can be used as a variant signature set for this specific clade. Using ancestral reconstruction methods, we compared the most recent common ancestor (MRCA) of the Marajó clade with ERR2815574, herein used as the outgroup isolate (Fig. 3). Using this approach, we detected 28 SNPs occurring between the two MRCA nodes of the phylogenetic tree.

Ideal clade-specific SNPs were defined as synonymous variants occurring in essential genes, which simultaneously decreases the likelihood of homoplasy driven by convergent evolution while ensuring that the region associated with the variant is conserved. Eight out of the 28 SNPs were found to be intragenic and synonymous and, of these, 3 were found to occur at genes know to be essential in *M. tuberculosis* H37Rv^22^. To validate this specific set, the 3 402 *M. bovis* publicly available genomes were re-screened for the initially identified SNPs. All 28 SNPs were found to occur only among the *M. bovis* isolates from Marajó Island with SNPs 2718745 T, 2830430 C and 3858304 C (*M. bovis* AF2122/97) considered as robust clade-specific SNPs for the Marajó strain (Table 3).

**Table 3:** Clade-specific SNPs detected for the Marajo strain along with affected genes, functional effect and affected gene product. All strain-specific SNPs are listed with positioning relative to the genome of *M. tuberculosis* H37Rv and *M. bovis* AF2122/97 with synonymous variants occurring at essential genes highlighted in bold.

Genomic Position (*M. tuberculosis* H37Rv)^a^	Genomic Position (*M. bovis* AF2122/97)^b^	ORF^c^	Gene Name	Reference allele	Altered allele	Mutation	Variant Type	Gene Essentiality^d^	Gene Product
463574	464591	Rv0386/Mb0393	—	T	C	T164C	missense	Non-essential	Probable transcriptional regulatory protein (probably LuxR/UhpA-family)
473386	474405	Rv0393/Mb0399	—	T	C	T606C	synonymous	Non-essential	Conserved 13e12 repeat family protein
770659	772428	Rv0671/Mb0690	*lpqP*	G	A	G78A	synonymous	Non-essential	Possible conserved lipoprotein LpqP
938123	938878	Rv0842/Mb0865	—	C	T	C12T	synonymous	Non-essential	Probable conserved integral membrane protein
990495	990960	Rv0890c/Mb0914c	—	T	C	A2102G	missense	Non-essential	Probable transcriptional regulatory protein (probably LuxR-family)
1067162	1067629	Rv0955/Mb0980	—	T	C	T1085C	missense	Non-essential^e^	Probable conserved integral membrane protein
1078797	1079264	Rv0969/Mb0994	*ctpV*	G	T	G55T	stop	Non-essential	Probable metal cation transporter p-type ATPase CtpV
1192229	1193617	Intergenic	—	A	G	—	—	—	—
1394348	1395620	Rv1250/Mb1282	—	T	G	T170G	missense	Non-essential	Probable drug-transport integral membrane protein
1513242	1515686	Rv1348/Mb1383	*irtA*	A	G	A196G	missense	Essential	Iron-regulated transporter IrtA
1529907	1532351	Rv1358/Mb1393	—	C	A	C3296A	missense	Non-essential	Probable transcriptional regulatory protein
2245157	2228483	Intergenic	—	C	A	—	—	—	—
2582546	2564552	Intergenic	—	T	C	—	—	—	—
2585942	2567948	Rv2314c/Mb2341c	—	A	G	T1349C	missense	Non-essential	Conserved protein
2744419	2716993	Rv2444c/Mb2471c	*rne*	C	T	G566A	missense	Essential	Possible ribonuclease E Rne
**2746171**	**2718745**	**Rv2447c/Mb2474c**	***folC***	**C**	**T**	**G1428A**	**synonymous**	**Essential**	**Probable folylpolyglutamate synthase protein folC (folylpoly-gamma-glutamate synthetase)**
**2859222**	**2830430**	**Rv2534c/Mb2563c**	***efp***	**G**	**C**	**C69G**	**synonymous**	**Essential**	**Probable elongation factor p Efp**
3027938	2988986	Rv2714/Mb2733	—	A	G	A874G	missense	Non-essential	Conserved alanine and leucine rich protein
3100631	3061592	Rv2791c/Mb2814c	—	T	G	A951C	synonymous	Non-essential	Probable transposase
3107666	3068627	Intergenic	—	C	G	—	—	—	—
3284440	3245425	Rv2941/Mb2966	*fadD28*	G	A	G1106A	missense	Non-essential	Fatty-acid-amp ligase fadd28 (fatty-acid-amp synthetase) (fatty-acid-amp synthase)
3479333	3440293	Rv3111/Mb3138	*moaC1*	A	G	A163G	missense	Non-essential^e^	Probable molybdenum cofactor biosynthesis protein c MoaC1
3600623	3559632	Rv3224/Mb3251	—	A	C	A773C	missense	Non-essential	Possible iron-regulated short-chain dehydrogenase/reductase
3606612	3565621	Rv3229c/Mb3258c	*desA3*	G	A	C423T	synonymous	Non-essential^e^	Possible linoleoyl-coa desaturase (delta(6)-desaturase)
3859073	3811188	Rv3439c/Mb3469c	—	G	T	C590A	missense	Non-essential	Conserved hypothetical alanine and proline rich protein
**3908268**	**3858304**	**Rv3490/Mb3520**	***otsA***	**T**	**C**	**T33C**	**synonymous**	**Essential**	**Alpha, alpha-trehalose-phosphate synthase**
4142051	4083811	Rv3699/Mb3725	—	A	C	A8C	missense	Non-essential	Conserved protein
4357167	4297899	Rv3878/Mb3908	*espJ*	T	C	T475C	missense	Non-essential	Esx-1 secretion-associated protein EspJ

^a^Relative to the genome position of *M. tuberculosis* H37Rv, GenBank Accession NC_000962.3;

^b^Relative to the genome position of *M. bovis* AF2122/97, GenBank Accession NC_002945.4;

^c^Open Reading Frame;

^d^Gene Essentiality according to DeJesus *et al*. (2017) using saturated Himar1 transposon libraries^22^;

^e^Non-essential gene by DeJesus *et al*.^22^, essential gene by Sassetti *et al*.^52^ and Griffin *et al*.^53^.

## Discussion

Livestock production is of the utmost economic importance in Brazil and is the mainstay of most inhabitants in the Marajó Island. The latter is the largest fluviomarine island in the world and its barriers to gene flow are highly relevant in the understanding of microbial biodiversity in socioeconomically relevant diseases, such as TB, and of the adaptation of local strains to specific ecological niches^23^. Over recent years, several aspects of the Marajó Island in the State of Pará has motivated special concern regarding bTB: the region is isolated from the mainland and notification of human TB by *M. bovis* is inexistent along with a very close contact of humans and animals (buffalo and cattle).

Two unusual spoligotypes (SB0822/SIT997 and SB0855/SIT986) of rare occurrence in Brazil were the only two profiles found among the 18 animals whose isolates were analyzed. This prompts us to a scenario of high strain endemicity and suggestive of a low, if any, strain flow from outside of this insular system. Moreover, 24-loci MIRU-VNTR typing yielded five profiles and three clusters were detected, yielding a clustering rate of 90%, and was unable to discriminate the single SB0855/SIT986 strain from most SB0822/SIT997 isolates. This preliminary data suggested that the single SB0855/SIT986 strain descends from the SB0822/SIT997 isolates and it also suggests that bTB epidemiology in this setting is dominated by a single strain undergoing clonal expansion across different herds of cattle and buffalos.

The SB0855/SIT986 profile is an uncommon one but it has been previously detected in European countries, such as France, Germany, Spain and Belgium. On the other hand, the SB0822/SIT986 profile is not as uncommon and, according to SITVIT2 and *Mycobacterium bovis* Spoligotyping Database (Mbovis.org), it shows widespread distribution across Europe, South America and North Africa with increased incidence in France. This latter profile has been in fact detected in Southeast Brazil (Minas Gerais and Espírito Santo states) but at a low prevalence when compared with other spoligotyping profiles^24^.

A possible link to France can be speculated since it has been proposed that bTB in South America has been introduced via cattle importation from Europe^25,26^. However, an alternative origin is also plausible since in the end of the 19^th^ century a ship taking buffalos from India to the French Guyana sank near the Marajó coast with the surviving animals becoming well adapted to the island^27^. Before this event, no records of water buffalos exist in this setting.

A more resolved phylogenetic scenario was obtained using WGS of 17 isolates from animals. Upon examining the overall distribution of pairwise SNP distances, and considering the dominance of a single spoligotype profile, we have obtained a broad SNP distance distribution indicating many missing links across the transmission chains and a large timeframe for *M. bovis* evolution in the area.

The genomic findings do highlight the limitations of 24-loci MIRU-VNTR in this specific setting where this highly prevalent SB0822/SIT997 strain already underwent significant diversification on a genome-wide scale. A total of three genomic clusters were identified, two of which encompassing three isolates and the remaining genomic cluster being composed of two clinical isolates for a total of eight clustered isolates (47%). Each cluster was found to be restricted to the same city and animal species which, *per se*, suggests that transmission is occurring at multiple geographical points with unapparent dissemination to other animal herds. While the genomic clusters herein detected are restricted to animals of the same species (two involving cattle and one involving buffalos) the tree topology is consistent with an evolutionary history marked by cross-species strain dissemination at multiple points in time since a specific sub-lineage to cattle or buffalos is not observed. Though the present study failed to detect recent transmission between different species, we hypothesize that these transmission events may well occur but at a lower rate since the phylogenetic structure of the clade does support it. Moreover, comparison with a global genomic dataset confirmed the uniqueness and genomic distinctiveness of the *M. bovis* Marajó strain herein described. Although the Marajó strains comprise a monophyletic clade, these strains are not classifiable in a known clonal complex but do in fact form a parallel branch with the European 2 strains while showing a high genetic differentiation from these. *M. bovis* European 2 strains are more prevalent in the Iberian Peninsula, are present at low frequencies in France and Italy, and are absent from the British Isles^18^. The phylogenetic history and multivariate populational genetics analysis show that isolates detected in Germany and USA are genetically closer, arguing mostly in favor of the emergence of this strain due to cattle importation from Europe with later dissemination across Buffalo herds.

Although the samples included in the study originated from the only abattoir with veterinary inspection in the island and the animals included were sourced from multiple sites, a limitation of this study is its sample size which may have hampered the detection of rare inter-species transmission events. This limitation might be related with the fact that only samples from animals displaying anatomopathological abnormalities or lesions suggestive of bTB were included in the study. A study conducted in South Brazil (Santa Catarina State) revealed that in *M. bovis* cattle positive for tuberculin/PPD antigen, only 8% of the animals showed clinical signs^28^.

To enable a rapid molecular screening and tracing of this unusual strain across multiple settings, we sought to identify strain-specific SNPs that can inform rapid molecular assays. The genomic SNP distance between the Marajó isolates and other isolates bearing the same spoligotyping profile but isolated in France revealed that spoligotyping is not an adequate marker since the strains from France and Marajó were phylogenetically located on distinct branches of the *M. bovis* phylogenetic tree, more than 413 SNPs apart. Given the clonal population structure of the MTBC species, SNP markers pose as attractive candidates for strain differentiation^29^. We were able to identify 28 potential SNPs that can be used as a barcode for these strains. Moreover, a total of three SNPs comprised synonymous intragenic SNPs located in genes classified as essential in M. tuberculosis H37Rv by saturated Himar1 transposon libraries^22^, which decreases the probability that the associated regions are lost due to further genome downsizing. The latter is a major mode of genome diversification throughout the evolutionary history of the MTBC^30^.

This set of specific SNPs identified in this study therefore has the potential to be incorporated in molecular screening strategies aimed at detecting the Marajó *M. bovis* strain at a global level using *in silico* approaches or, at the state or national level in *M. bovis* strain biobanks to further evaluate the dissemination of this clade to other regions in Brazil and to assess the risk to human health. This type of approach, using allele-specific PCR amplification has been successfully used to evaluate the dissemination of specific strains outside its endemic region, understand the transmission dynamics at the cross-border level or enable the evaluation of TB transmission in settings without routine or universal molecular typing^31^.

Cultural aspects associated with living conditions in the Marajó such as drinking raw milk or eating dairy products produced from raw unpasteurized milk pose a specific threat to human health. So far, *M. bovis* infection in humans has not been reported or detected in the island population or in the state of Pará, however, this could be due to a combination of a low detection rate, inexistent laboratory confirmation of clinically suspected cases or identification at the species-level and, lack of routine strain typing at the state-level.

In conclusion, this study combines classical typing methods along with genome-wide sequence data in the identification and delineation of a unique *M. bovis* strain, that is herein designated as the Marajó strain. As the real magnitude and impact of bTB as a zoonotic disease in northern Brazil remains unknown and there is a considerable and underestimated bTB risk to humans in the Marajó Island, the study provides information on isolates from the livestock sectors in Brazil and on the origin of *M. bovis* strains. Such information is essential in the development and implementation of future bTB control strategies for the north of Brazil and, the molecular markers identified will aid in the development of rapid molecular assays that can be deployed at multiple settings and assess the specific risk posed by this strain to human health and food safety.

## Materials and Methods

### Data and sample collection

The present study includes a total of 20 *M. bovis* isolates from 18 different animals slaughtered at the Soure municipal abattoir, Marajó Island, in North of Brazil. These isolates ensued from the screening of 48 cattle and buffalo, slaughtered for meat production purpose, presenting TB-like lesions, from October 2014 to December 2015. Briefly, 13 isolates were obtained from nine cattle and the remaining nine isolates from buffalos. Different points of origin for these animals were detected within the island: six animals from Chaves, 10 from Soure, one from Santa Cruz do Arari and one from Cachoeira do Arari (Table 1). The samples were obtained during official post-mortem routine inspection performed by the veterinarian technical manager of the municipal abattoir and this study was submitted for ethical review by the Animal Use Ethics Committee of the State University of Pará, which issued an opinion waiver certificate in accordance with Brazilian Law 11794 (October 08, 2008).

### Isolation and identification of mycobacterium bovis

Tissue sample was macerated and decontaminated with SDS(3%) and NaOH(1%), neutralized with bromothymol blue solution and spread evenly onto Löwenstein-Jensen media slants supplemented with pyruvate or with pyruvate and p-nitrobenzoic acid. Screening for the presence of acid-fast bacilli was performed by Ziehl-Neelsen staining and microscopy. Isolates displaying a slow-growth rate incapable of growing on media supplemented with p-nitrobenzoic acid were presumptively identified as MTBC isolates. Molecular confirmation was carried out by PCR amplification and partial sequencing of the *hsp65* gene as previously described^32^. DNA extraction was carried out using a phenol-chloroform extraction method^33^.

### Spoligotyping

Spoligotyping was performed as previously proposed by Kamerbeek *et al*.^34^ and implemented on a Luminex 200™ platform automated system (Luminex Corp, Austin, TX) using polystyrene microbeads^35^. The result was analyzed in the SITVIT2 (http://www.pasteur-guadeloupe.fr:8081/SITVIT2/) and *Mycobacterium bovis* Spoligotyping Database (https://www.mbovis.org/).

### Mycobacterial interspersed repetitive units-variable number of tandem repeat (MIRU-VNTR) typing

PCR amplification of DNA for MIRU-VNTR typing was performed for a set of 24 tandem repeat loci as previously described^36^. PCR reactions were performed in using GoTaq^®^ DNA Polymerase following manufacturer’s instructions (Promega Corporation, Wisconsin, USA). Amplicon sizing and allelic determination was performed by agarose gel electrophoresis on a 2% (w/v) agarose gel. A MIRU-VNTR cluster was defined as a group of two or more isolates sharing identical profiles.

### Whole genome sequencing

DNA quantification was performed using the Qubit™ dsDNA HS Assay Kit (Thermo Fisher Scientific, Waltham, USA) and the Agilent High Sensitivity DNA Kit (Agilent, California, USA). WGS of *M. bovis* isolates was carried out on a NextSeq instrument (Illumina, San Diego, CA) using a 2 × 150 paired-end chemistry and the Nextera XT library preparation kit (Illumina, San Diego, CA).

### Phylogenomic analysis

Quality trimming and filtering of Illumina reads were performed using the Trimmomatic v0.36 by cutting reads whose average quality falls below an average PHRED score of 20 in a 4 bp sliding window and retaining reads with minimum length of 36 bp^37^. Filtered reads were mapped against the *M. bovis* AF2122/97 genome (GenBank Accession NC_002945.4)^38^ using the Burrows Wheeler Aligner tool (BWA-MEM algorithm)^39^. Mapping statistics were obtained using Qualimap^40^. SAMtools and GATK were used for variant calling and only concordant variants were retained for downstream analysis^41,42^.

High-quality SNPs were extracted and concatenated into a single DNA pseudo-molecule. A minimum site coverage of 20 reads were used and variant sites retained only if the majority nucleotide reached a relative coverage depth of 75%. A missing call was assigned if a base call did not met the previous criteria and samples or SNP sites having an excess of 10% missing calls were excluded as to remove heterogenic and low coverage sites^43^. SNP positions falling in mycobacterial PPE/PE genes and repeat regions were removed from the final alignment. The final dataset across 17 isolates and *M. bovis* AF2122/97 (reference) encompassed a total of 1 773 high-quality SNPs, 1 559 (87.9%) of which had no missing genotypes (core SNP sites). Pairwise SNP distance was evaluated using snp-dists (https://github.com/tseemann/snp-dists).

A maximum-likelihood phylogenetic tree rooted on *Mycobacterium caprae* EPDC01^44^ was created with SeaView v.4 software implementing PhyML using a Generalized Time Reversible (GTR) model without gamma variation and invariable sites^45^. Tree topology was optimized by searching the tree space using the Subtree Pruning and Regrafting (SPR) and, the Nearest Neighborhood Interchange (NNI) methods. Branch reliability was estimated using the approximate Likelihood Ratio Test (aLRT)^46^.

The resulting tree was annotated and rooted using the Interactive Tree of Life v5.3 (iTOL) online tool (available at https://itol.embl.de/)^47^. Ancestral sequence reconstruction was carried out using the package phangorn in R. The goeBURST/Phyloviz tool (available at https://online2.phyloviz.net) was used to identify genomic transmission clusters using a 5 SNP threshold and to generate minimum spanning trees.

Additionally, variants associated with drug resistance were detected using TB-Profiler v2.6.1 (https://github.com/jodyphelan/TBProfiler)^16,48^.

### Comparison with a global *M. bovis* dataset

Genomic variant data was compared to a global collection of 3 402 *M. bovis* genomes publicly available on the European Nucleotide Archive (ENA; Supplementary Table S1) until December 2018 (initial: 3 590, 188 isolates excluded due to low coverage of sequence data or inability to generate a spoligotyping profile using SpoTyping). This genome collection is part of a *M. bovis* genomic variant dataset available at iMed.ULisboa and, is composed of variant call data and individual site mapping statistics obtained through the Snippy mapping pipeline using *M. bovis* AF2122/97 (GenBank Accession NC_002945.4) as reference genome^49^. Two additional *M. bovis* genomes from ANSES (*Agence Nationale de Sécurité Sanitaire de l’Alimentation, de l’Environnement et du Travail*), Maisons-Alfort, France, sharing similar spoligotyping profiles were included (ENA study accession SRP161870). *SpoTyping* was used to determine *in silico* spoligotyping profiles for all isolates^50^. If available, metadata on the year of isolation and country of origin was extracted from sample associated XML files available at ENA. Genome-wide high-quality SNPs (Total: 42 419 segregating sites) were extracted from VCF files and coverage-validated using the same parameters described above across the entire genomic dataset, including the Marajó isolates. A phylogenetic tree was constructed for a final dataset composed of 257 *M. bovis* isolates which included the 17 isolates recovered at the Marajó Island, the two additional *M. bovis* isolates from ANSES and 237 *M. bovis* genomes of isolates from more than 16 countries, representative of all spoligotypes (SB type, Mbovis.org) available across the 3 402 *M. bovis* isolates whose genome was publicly available. For this final dataset, high-quality SNPs were obtained as described for the 17 Marajó *M. bovis* isolates totalling 20 103 high-quality SNP sites, of which 11 544 (57.4%) had no missing genotypes (core SNPs).

### Population genetics

Populational genetic multivariate analysis was carried out in R using the adegenet package. Principal Component Analysis (PCA) and Principal Coordinate Analysis (PCoA) was carried out for the 257 *M. bovis* dataset using the 11 544 coreSNPs identified across this sample and along three principal components. Inter-populational comparison and differentiation analysis was done by considering the previously described Clonal Complexes (European 1–2 and African 1–2) and assigning each isolate to one of these populations based on specific genomic markers^18,20–22^. Isolates deemed unclassifiable as per this scheme were assigned to the Unclassified population and isolates recovered at the Marajó Island were, given its monophyletic nature, assigned to the Marajo population. Pairwise F_ST_ distances between populations was computed as a metric of populational paiwise differentiation using R along with the dartR and StAMPP packages implementing the method described by Weir and Cockerham (1984) with 1 000 bootstraps^51^.

### Accession numbers

Study accession ERP116404.

## Supplementary information

Supplementary information.

Supplementary information2.

## Data Availability

Raw sequence data has been submitted to the ENA under the study accession ERP116404. Sample information along with run accession numbers for publicly available genomes used in this study can be found at the Supplementary Table S1.
