# Supplementary material for: Phylogenomic Perspective on a Unique Mycobacterium bovis Clade Dominating Bovine Tuberculosis Infections among Cattle and Buffalos in Northern Brazil
Source: Sci Rep. 2020 Feb 4;10:1747. doi: 10.1038/s41598-020-58398-5 (PMC7000724; doi:10.1038/s41598-020-58398-5)
Supplement: Supplementary file 1 — Supplementary information. [file 41598_2020_58398_MOESM1_ESM.pdf]

Supplementary Information

Phylogenomic Perspective on a Unique *Mycobacterium bovis* Clade Dominating Bovine Tuberculosis Infections among Cattle and Buffalos in Northern Brazil

Marília Lima da Conceição[1,2,3#], Emilyn Costa Conceição[4,5#], Ismari Perini Furlaneto[1], Sandro Patroca da Silva[2], Arthur Emil dos Santos Guimarães[1,2], Pedro Gomes[3], María Laura Boschiroli[6], Lorraine Michelet[6], Thomas Andreas Kohl[7,8], Katharina Kranzer[9,10], Loreno da Costa Francez[11], Luana Nepomuceno Gondim Costa Lima[2], Isabel Portugal[3], João Perdigão[3]*, Karla Valéria Batista Lima[1,2]*.

[1] State University of Pará, Belém, Brazil.

[2] Evandro Chagas Institute, Anandideua, Brazil.

[3] Research Institute for Medicines (iMed.ULisboa), Faculty of Pharmacy, Universidade de Lisboa, Lisbon, Portugal.

[4] Federal University of Rio de Janeiro, Rio de Janeiro, Brazil.

[5] Oswald Cruz Foundation, Rio de Janeiro, Brazil.

[6] French Agency for Food, Environmental and Occupational Health and Safety, Maisons-Alfort, France.

[7] Leibniz Research Center Borstel, Borstel, Germany.

[8] German Center for Infection Research (DZIF), Heidelberg, Germany.

[9] Research Center Borstel Leibniz Lung Center, National Reference Center for Mycobacteria, Borstel, Germany.

[10] London School of Hygiene and Tropical Medicine, London, UK London School of Hygiene and Tropical Medicine, Clinical Research Department, London, UK.

[11] Federal Rural University of Amazon, Belém, Brazil.

[#] These authors are joint first authors

***** Both authors share senior authorship

Corresponding authors:
Marília Lima da Conceição
Instituto Evandro Chagas, Rodovia BR-316, 67030-000 Anandideua, Pará, Brazil
Phone: +55 (91)32142124, E-mail: marilimadc@msn.com

João Perdigão
Instituto de Investigação do Medicamento, Faculdade de Farmácia, Universidade de Lisboa, Av. Prof. Gama Pinto, 1649-003 Lisboa, Portugal
Phone: +351 2179464440, Fax: +351 217934212, E-mail: jperdigao@ff.ulisboa.pt

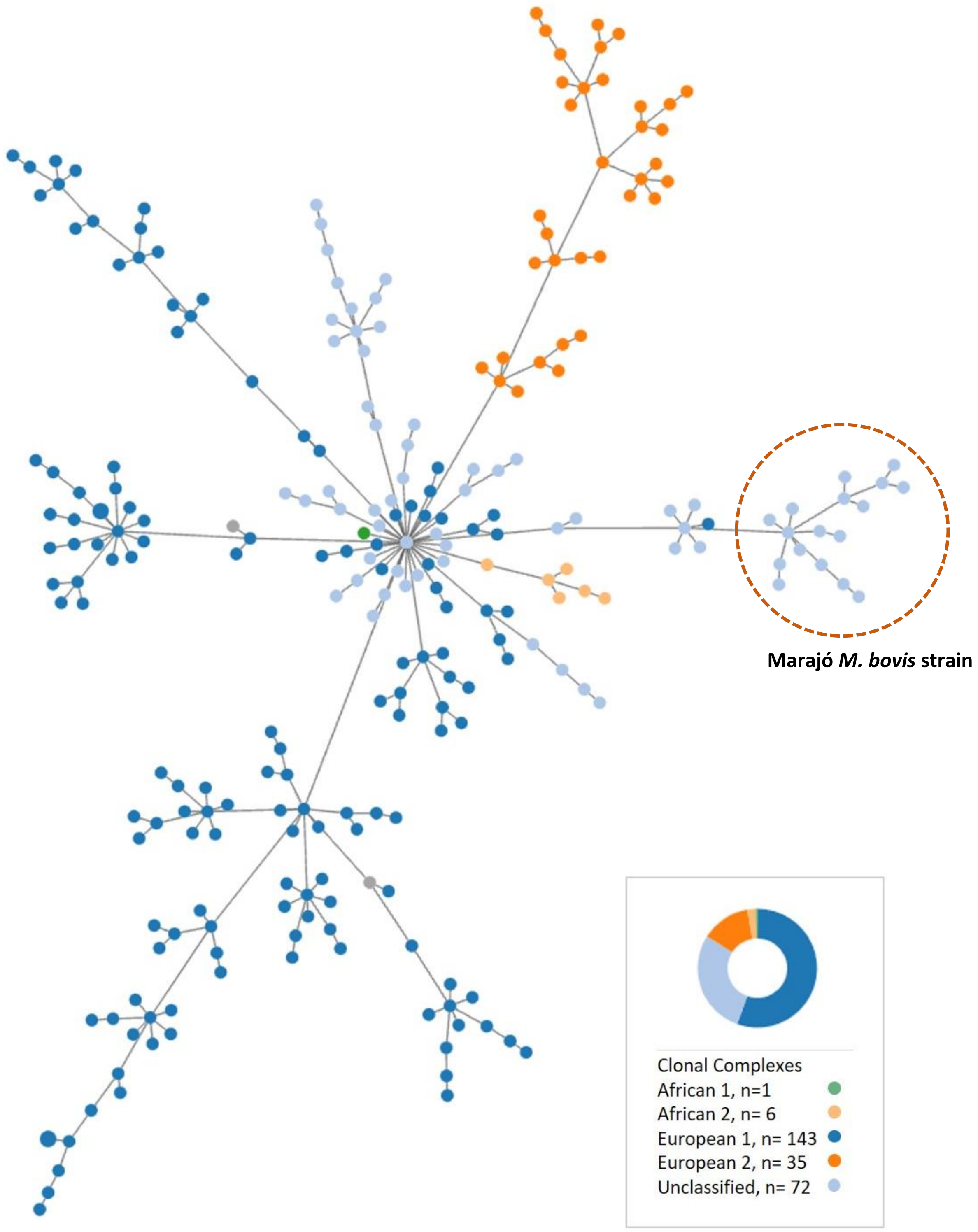

Supplementary Figure S1 – Minimum Spanning Tree of 257 *M. bovis* isolates, including 17 M. bovis isolated from buffalos and cattle in the Marajó Island. The Minimum Spanning Tree was constructed with the goeBURST algorithm using the pairwise distance method as implemented in Phyloviz (http://online2.phyloviz.net/index) and based on 20 103 SNPs. Nodes are coloured by Clonal Complex. Both European 2 and African 2 isolates comprise distinct monophyletic branches, contrarily to the European 1, which, using this algorithm does not form a single monophyletic subtree. The Marajó strains are highlighted on the tree forming a separate branch.

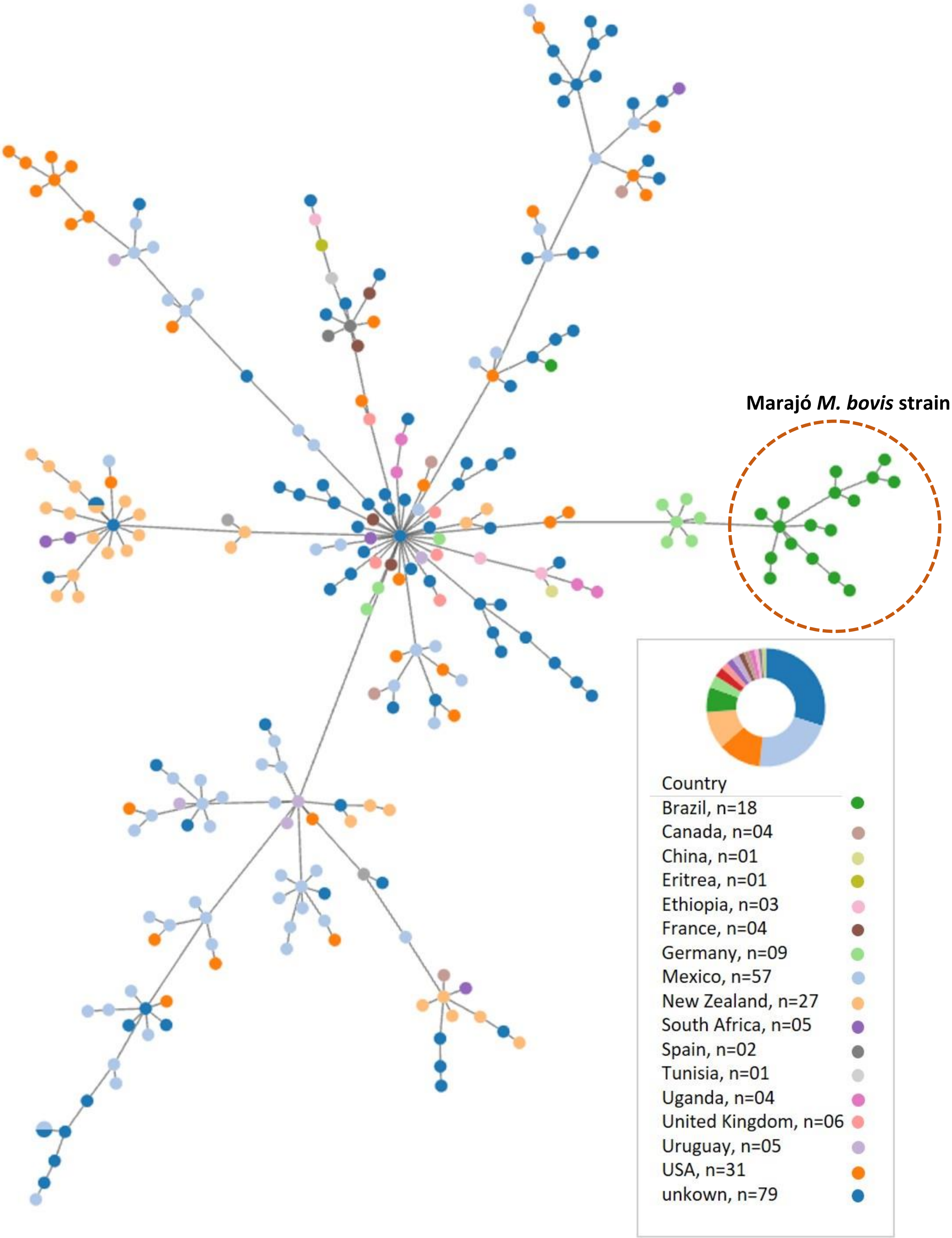

Supplementary Figure S2 – Minimum Spanning Tree of 257 *M. bovis* isolates, including 17 M. bovis isolated from buffalos and cattle in the Marajó Island. The Minimum Spanning Tree was constructed with the goeBURST algorithm using the pairwise distance method as implemented in Phyloviz (http://online2.phyloviz.net/index) and based on 20 103 SNPs. Nodes are coloured by country of isolation. The Marajó strains are highlighted on the tree forming a separate branch, distancing at least 79 SNPs from the nearest *M. bovis* isolate (Germany)
